# A cross-sectional study of physical activity and sedentary behaviours in a Caribbean population: combining objective and questionnaire data to guide future interventions

**DOI:** 10.1186/s12889-016-3689-2

**Published:** 2016-10-01

**Authors:** Christina Howitt, Soren Brage, Ian R. Hambleton, Kate Westgate, T. Alafia Samuels, Angela MC Rose, Nigel Unwin

**Affiliations:** 1 Chronic Disease Research Centre, Caribbean Institute for Health Research, The University of the West Indies, Jemmott’s Lane, Bridgetown, West Indies Barbados; 2 MRC Epidemiology Unit, University of Cambridge, Cambridge, UK

**Keywords:** Physical activity, Sedentary behaviour, Physical activity questionnaire, Combined sensing, Barbados

## Abstract

**Background:**

Current understanding of population physical activity (PA) levels and sedentary behaviour in developing countries is limited, and based primarily on self-report. We described PA levels using objective and self-report methods in a developing country population.

**Methods:**

PA was assessed in a cross-sectional, representative sample of the population of Barbados (25–54 years), using a validated questionnaire (RPAQ) and individually calibrated combined heart rate and movement sensing monitors. The RPAQ collects information on recalled activity in 4 domains: home, work, transport, and leisure. Physical inactivity was defined according to World Health Organization (WHO) guidelines; sedentary lifestyle was defined as being sedentary for 8 h or more daily; PA overestimation was defined as perceiving activity to be sufficient, when classified as ‘inactive’ by objective measurement.

**Results:**

According to objective estimates, 90.5 % (95 % CI: 83.3,94.7) of women and 58.9 % (48.4,68.7) of men did not accumulate sufficient activity to meet WHO minimum recommendations. Overall, 50.7 % (43.3,58.1) of the population was sedentary for 8 h or more each day, and 60.1 % (52.8,66.9) overestimated their activity levels. The prevalence of inactivity was underestimated by self-report in both genders by 28 percentage points (95 % CI: 18,38), but the accuracy of reporting differed by age group, education level, occupational grade, and overweight/obesity status. Low PA was greater in more socially privileged groups: higher educational level and higher occupational grade were both associated with less objectively measured PA and more sedentary time. Variation in domain-specific self-reported physical activity energy expenditure (PAEE) by educational attainment was observed: higher education level was associated with more leisure activity and less occupational activity. Occupational PA was the main driver of PAEE for women and men according to self-report, contributing 57 % (95 % CI: 52,61). The most popular leisure activities for both genders were walking and gardening.

**Conclusions:**

The use of both objective and self-report methods to assess PA and sedentary behaviour provides important complementary information to guide public health programmes. Our results emphasize the urgent need to increase PA and reduce sedentary time in this developing country population. Women and those with higher social economic position are particularly at risk from low levels of physical activity.

**Electronic supplementary material:**

The online version of this article (doi:10.1186/s12889-016-3689-2) contains supplementary material, which is available to authorized users.

## Background

Physical inactivity is estimated to have caused 9 % of premature mortality in 2008, around 5.3 million deaths [1]. The World Health Organization’s (WHO) global action plan on non-communicable diseases (NCDs) commits countries to reducing physical inactivity by 10 % by 2025 from its level in 2010 [2]. In order to know whether this target has been met, countries will require robust surveillance data to quantify population levels of inactivity and to assess change over time. Ideally, surveillance data should be available by population subgroup, to help identify inequalities and guide intervention planning.

Due to their low cost and low participant burden, questionnaires are the most commonly used method to assess population physical activity (PA) levels and patterns, including the prevalence of inactivity. However, PA questionnaires are prone to bias, including social desirability and recall bias [3]. Objective methods provide more valid and precise measures of PA, and remove the difficulties associated with recalling habitual activities [4]. It is now recognised that self-reported quantitative estimates of PA show poor agreement with objective estimates [5], but provide information on the context and types of activities that complements objective measures [4]. Objective measures of PA are increasingly being incorporated into national surveillance systems for non-communicable disease (NCD) risk factors, although cost has mainly limited uptake to developed countries [6]. Developing countries remain largely dependent on questionnaires to determine population levels of physical inactivity [6], even though using this potentially biased approach could have undesirable implications for public health policy aimed at increasing physical activity. Ideally, PA surveillance would utilise both techniques to provide complementary information to guide interventions, as implemented, for example, in the US [7] and the UK [8].

The Caribbean region is comprised mainly of developing countries [9]. The majority of the burden of disease is due to NCDs, including diabetes, cardiovascular disease and cancers [10]. Despite this, information on the behaviours that contribute to the high prevalence of these diseases, including PA, is sparse. Recently, through the efforts of the Global Observatory for Physical Activity, standardised data on the prevalence of physical inactivity, collected by questionnaire, have been collated for several countries in the region [11]. These data highlight substantial levels of inactivity, and consistent within-country gender inequalities, with women more inactive than men [11]. However, these estimates, and to our knowledge, all other national data on PA in the Caribbean [12, 13], have been derived from questionnaires. Using objective measures alongside questionnaires to improve our understanding of PA and sedentary behaviour would facilitate the design of interventions tailored to these and similar populations.

Barbados is an independent Caribbean country with a population in 2010 of around 280,000 [14], and in which 80 % of deaths are due to NCDs [15]. Although it is classified by the World Bank as a high income country [16], it is classified by the International Monetary Fund (IMF) as a developing country [9] and is a member of the United Nations Conference of Small Island Developing States (SIDS) [17]. Our main objective in this paper is to describe PA and sedentary behaviour in young to middle-aged Barbadian adults, using both objective and self-reported measures to estimate the prevalence and provide the context of these behaviours. In doing so, we present the most complete picture of PA and sedentary behaviour in an adult Caribbean population to date. We also quantify the underestimation of physical inactivity introduced by questionnaire-based assessment, and explore whether the extent of this bias is large enough to influence public health decision-making. This would have important implications for the many developing country settings with PA assessment limited to self-reported measurement.

## Methods

### Study population

The Health of the Nation (HotN) survey was a cross-sectional survey of the Barbadian population aged 25 years and over, which aimed to provide estimates of the prevalence and social distribution of NCD risk factors. Data were collected for 1234 participants on health conditions and behaviours (including questionnaire-assessed PA), as well as sociodemographic information. The survey has been described in detail elsewhere [18]. An age-restricted sub-sample (25–54 years; *n* = 527) of participants who completed HotN was selected for objective PA measurement. We decided to focus limited resources for PA measurement on this younger age group, because of the opportunity to intervene and change behaviour before the majority of NCDs develop.

Written informed consent was obtained from all participants. The study was approved by the Research Ethics Committee of the University of the West Indies, Cave Hill/Barbados Ministry of Health.

### Assessment of physical activity

#### Physical activity variables

The PA and sedentary behaviour variables used in this analysis are defined in Table 1. Physical activity intensity variables were derived using the standard definition of 1 metabolic equivalent (MET) as 3.5 ml of O_2_ per kg per minute [19].

**Table 1:** Definitions of Physical activity and sedentary behaviour variables

Variable		Definition
Physical inactivity	Prevalence (%)	Does not meet WHO minimum recommendations for physical activity, i.e. at least 150 min of MVPA per week accrued in bouts of at least 10 min [29]
Sedentary lifestyle	Prevalence (%)	Spends 8 h or more sedentary each day, excluding sleep [30]
Overestimator	Prevalence (%)	Perceives themselves as ‘active’ or ‘extremely active’ but does not meet WHO minimum guidelines for activity according to objective estimate
Physical activity energy expenditure (PAEE)	(kJ/kg/day)	The modifiable component of daily total energy expenditure derived from all activities
Sedentary time	(hours/day)	Time spent at ≤1.5 MET [29]
Light physical activity (LPA)	(hours/day)	Time spent between 1.5 and 3 MET [29]
Moderate-to-vigorous physical activity (MVPA)	(mins/day)	Time spent at ≥3 MET [29]

#### Self-reported activity: RPAQ

Physical activity was self-reported using the Recent Physical Activity Questionnaire (RPAQ), which was originally validated for use in the UK [20] and subsequently in other European countries [21]. The RPAQ was administered as part of the main HotN survey. The RPAQ assesses PA over the past 4 weeks in the following four domains: at home, during transport, at work, and in leisure time. Prior to data collection, a pilot study of the RPAQ was conducted on a sample of 20 Barbadian adults, aged 25 years and older. Minor changes were required to the leisure section to reflect the different activities carried out in this population. The pilot study also showed that very few people in Barbados are aware of the distance between their home and work. We therefore collected the participants’ home and work addresses, and used an online mapping service [22] to estimate the distance travelled. The modified RPAQ is available in the Additional files 1, 2, 3 and 4.

Physical activity summary variables were derived from the RPAQ using previously reported methodology [20]. Occupation was classified into 4 groups, which were assigned an assumed energy cost as follows: sedentary: 1.34 MET; standing: 1.62 MET; manual: 1.94 MET; and heavy manual: 2.11 MET [21].

The leisure section of the RPAQ lists 37 activities; participants indicated how often they carried out each activity and gave the average duration of each episode over the last 4 weeks. The activities were condensed into nine summary activities: swimming; walking; running; cycling; aerobics; gardening; racquet sports; water sports; and team sports. The constituent activities for each group are detailed in the Additional files 1, 2, 3 and 4.

#### Objective activity: combined heart rate and movement sensing

Physical activity was measured objectively using a combined heart rate and movement sensor (Actiheart; CamNtech Ltd, Cambridge, UK). Participants were asked to wear the monitor continuously for 7 days and to continue their normal activities. The monitor was initialised to record at 15 s epochs.

An 8-min incremental step test was used to calibrate the individual’s heart rate response to activity intensity [23]. Participants did not complete the step test if they were unwilling, if they reported being unable to walk unaided at a brisk pace for at least 10 min, or if they reported taking at least half the maximum daily dose of beta blocker medication. The step test was terminated if the participant could not keep up with the stepping protocol or if they asked to stop.

A Gaussian process regression method was applied to the heart rate data to handle potential measurement noise [24]. Free-living activity intensity (J/min/kg) was estimated from acceleration and individually calibrated heart rate [23] using a branched equation framework [25]. A group calibration equation was derived for this population using the valid step tests [23]. If individual calibration was not available, the group calibration equation was applied. Non-wear time was taken into account and adjusted for in order to minimise potential diurnal bias when summarising data into PAEE and time spent at different intensities [26]. The PAEE estimate has been validated during free-living against doubly-labelled water in both African [27] and European populations [28].

Time spent in MVPA was summarised in two ways. The first included all episodes of MVPA regardless of their duration. We also created a MVPA variable (MVPA_BOUT_) that excluded episodes with a duration of less than 10 min. In accordance with WHO guidelines [29], MVPA_BOUT_ was used to define physical inactivity, i.e. those who did not meet the minimum recommendations for PA.

There is no standard definition of sedentary lifestyle, but we chose the cutpoint based on a recent, large-scale study that demonstrated the association between sitting for 8 h or more every day and increased all-cause mortality [30]. In order to exclude sleep from sedentary time, the RPAQ-derived sleep time was subtracted from the objectively assessed total sedentary time.

### Perception of activity

Prior to the objective assessment, participants were asked to rate their own activity as either ‘extremely active’ , ‘moderately active’ or ‘not very active’. Perceived activity was compared to the objective measurements. We classified ‘overestimators’ as those who considered themselves extremely or moderately active, but were classified objectively as inactive according to WHO guidelines.

### Assessment of other risk factor and sociodemographic variables

Details of the methods for assessing risk factors and sociodemographic characteristics in the HotN survey have been published elsewhere [18]. Body mass index (BMI; kg/m^2^) was derived from height and weight measured using standard protocols and categorised as normal weight (<25 kg/m^2^), overweight (25–30 kg/m^2^), and obese (>30 kg/m^2^).

Maximum level of educational attainment and occupational grade were used in this analysis as indicators of socioeconomic position (SEP). Education was grouped into four levels as follows: level 1 had not completed secondary school; level 2 completed secondary school; level 3 had technical, trade or teacher education; and level 4 had university education (undergraduate and postgraduate). Occupation was collected as free text and then coded using the Barbados Standard Occupational Classification (BARSOC-89) [31], which is based on the 1988 International Standard Classification of Occupations (ISCO-88) [32]. BARSOC-89 contains nine major groups, which were collapsed to create three broad occupational categories: group 1 (routine/manual) consisted of skilled agricultural, craft/elementary workers, and machine operators; group 2 (intermediate) comprised technical, clerical and service employees; and group 3 (professional) consisted of managers and professionals.

### Statistical analyses

Descriptive statistics for the continuous PA variables (PAEE, sedentary time, time spent in LPA, time spent in MVPA) are presented as median and interquartile range (IQR) due to their skewed distribution.

The prevalence of physical inactivity (i.e. not meeting WHO minimum recommendations) and sedentary lifestyle (at least 8 h per day sedentary, excluding sleep) are presented as proportions and 95 %CIs. The difference in prevalence of physical inactivity between the self-report and the objective measure is presented with 95 % confidence intervals (CIs), and with the difference formally examined using McNemar’s test for paired data.

Associations between PA components and SEP indicators (education level and occupational grade) were examined using multivariable regression models. Due to the skewed distribution of the PA components, median regression was used, as recommended by McGreevy et al. [33].

Data were analysed using the Stata software package (version 13, StataCorp, College Station, Texas). All analyses were weighted to account for the sampling design and non-response, and to match the age and sex distribution of the Barbadian population according to the 2010 Barbados Population and Housing Census [14].

## Results

**Fig. 1:**
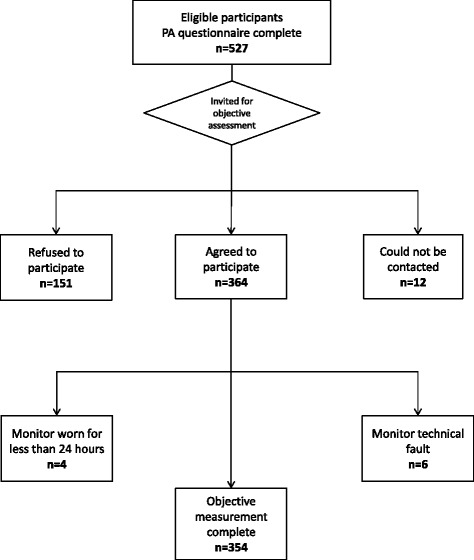
Flowchart of study recruitment

Of the 527 individuals invited to have an objective PA assessment, 364 participated, 151 refused, and 12 could not be contacted. The response rate for this study was 69 %. An additional 10 participants were excluded due to the monitor being worn for less than 24 h (*n* = 4) and monitor technical faults (*n* = 6). In total, 354 participants were included in this analysis. Recruitment for the study is summarised in Fig. 1. The median (IQR) wear time for the monitor was 166 (139, 169) hours in women and 167 (154, 172) hours in men. Valid step test data were obtained for 330 participants (93 %); group calibration equations were derived from these tests and were applied to the remainder of the sample. Median (IQR) time between the RPAQ and objective assessments was 114 (72, 173) days.

### Participant characteristics

Demographic, socioeconomic, and anthropometric characteristics of the sample are shown in Table 2. The prevalence of overweight was similar for both genders: 25.6 % (95 % CI: 19.6,32.7) in women and 33.0 % (25.0,42.2) in men. In contrast, the prevalence of obesity in women was statistically significantly higher than that in men: 49.5 % (42.8,56.3) vs. 20.5 % (14.0,29.0).

**Table 2:** Demographic, socioeconomic, anthropometric and physical activity characteristics of sample^a^ok

		Women (*n* = 216)		Men (*n* = 138)		Overall (*n* = 354)	
		%	95 % CI	%	95 % CI	%	95 % CI
Age							
25–39 years	(*n* = 130)	47.1	(40.0,54.4)	49.5	(39.2,59.8)	48.3	(42.3,54.3)
40–54 years	(*n* = 224)	52.9	(45.6,60)	50.5	(40.2,60.8)	51.7	(45.7,57.7)
Education							
Less than secondary school	(*n* = 28)	6.6	(4.3,9.8)	5.5	(2.3,12.6)	6.0	(3.8,9.4)
Secondary school completed	(*n* = 174)	52.5	(44.8,60.1)	43.2	(33.5,53.5)	48.0	(41.0,55.2)
Technical, trade or teacher	(*n* = 64)	19.7	(13.7,27.5)	26.1	(16.9,38)	22.8	(16.6,30.4)
University	(*n* = 88)	21.2	(14.8,29.4)	25.2	(17.1,35.5)	23.2	(17.1,30.6)
Occupational grade							
Routine/manual	(*n* = 83)	9.6	(6.3,14.5)	40.3	(30.6,50.8)	24.5	(19.3,30.4)
Intermediate	(*n* = 114)	41.6	(35.4,48)	21.0	(13.1,31.9)	31.6	(27.3,36.2)
Professional	(*n* = 58)	14.7	(10.3,20.5)	16.5	(10.1,25.8)	15.6	(11.4,21)
Not in employment	(*n* = 76)	29.6	(22.9,37.2)	11.5	(7,18.3)	20.8	(16.0,26.7)
Missing^b^	(*n* = 23)	4.5	(2.0,10.2)	10.8	(6.4,17.6)	7.5	(4.7,11.8)
BMI category							
Normal	(*n* = 113)	24.9	(19.3,31.4)	46.5	(35.6,57.7)	35.3	(29.0,42.2)
Overweight	(*n* = 113)	25.6	(19.6,32.7)	33.0	(25.0,42.2)	29.2	(23.8,35.3)
Obese	(*n* = 128)	49.5	(42.8,56.3)	20.5	(14.0,29)	35.5	(29.7,41.8)
Physical activity characteristics							
Objective inactive	(*n* = 177)	90.5	(83.3,94.7)	58.9	(48.4,68.7)	75.2	(68.7,80.8)
Objective sedentary	(*n* = 181)	57.1	(50.1,63.8)	43.9	(33,55.3)	50.7	(43.3,58.1)
Overestimator	(*n* = 211)	66.8	(57.1,75.3)	52.7	(43.6,61.7)	60.1	(52.8,66.9)

^a^Percentages are weighted to compensate for unequal probabilities of selection (selecting 1 individual from household), non-response, and to standardise to the age-sex distribution according to the latest population census

^b^Occupation provided as free text, but did not fit any code

Demographic, socioeconomic, and anthropometric characteristics of the complete HotN sample (aged 25–54; *n* = 704) are shown in Additional file 3: Table S1. There were no significant differences (with all 95 % CIs overlapping) in these characteristics between the HotN sample and the sample that completed the PA assessment.

### Objectively assessed physical activity and sedentary behaviour

The prevalence of objectively assessed inactivity and sedentary lifestyle are shown in Table 2. According to objective data, more women than men were inactive: 90.5 % (83.3,94.7) vs. 58.9 % (48.4,68.7). In contrast, there was not a significant difference in sedentary lifestyle between men and women, with the prevalence in both sexes combined being 50.7 % (95 % CI: 43.3,58.1).

Table 3 presents estimates of PA and sedentary behaviour derived from objective data. Median (IQR) PAEE was 36 (27,46) and 47 (34,63) kJ/kg/day for women and men, respectively. Median (IQR) time spent sedentary, in LPA, and MVPA was 8.3 (6.6,10.1) hours/day, 6.4 (5.2,8.0) hours/day, and 56.6 (38.2,97.4) minutes/day, respectively, amongst women; and 7.6 (5.6,9.5) hours/day, 6.8 (5.8,8.0) hours/day, and 91.6 (41.2,146.0) minutes/day, respectively, amongst men.

**Table 3:** Objectively assessed physical activity components in young to middle aged adult Barbadians

	PAEE (kJ/kg/day)		Sedentary time (hours/day)		LPA (hours/day)		MVPA (mins/day)	
	Median	IQR	Median	IQR	Median	IQR	Median	IQR
Overall	40.9	(31.7,53.3)	8.0	(6.2,9.8)	6.7	(5.5,8.0)	62.5	(40.2,130.3)
Sex								
Women	36.4	(26.7,45.9)	8.3	(6.6,10.1)	6.4	(5.2,8.0)	56.6	(38.2,97.4)
Men	47.2	(34.4,62.5)	7.6	(5.6,9.5)	6.8	(5.8,8.0)	91.6	(41.2,146)
Age group								
25–39 years	44.2	(32.9,55.3)	7.5	(5.6,9.6)	6.9	(5.7,8.2)	72.3	(40.5,143)
40–54 years	37.6	(29.6,52)	8.3	(6.7,10)	6.4	(5.4,7.6)	58.5	(39.8,112.4)

### Comparison of objective and self-reported estimates of physical activity

The prevalence of PA overestimation was similar in men and women (Table 2). Overall, 60.1 % (95 % CI: 52.8,66.9) overestimated their activity.

**Fig. 2:**
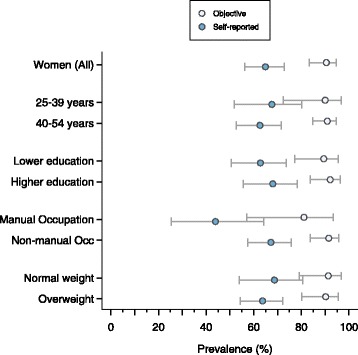
The prevalence of objective and self-reported physical inactivity in Barbadian women, stratified by sociodemographic group

**Fig. 3:**
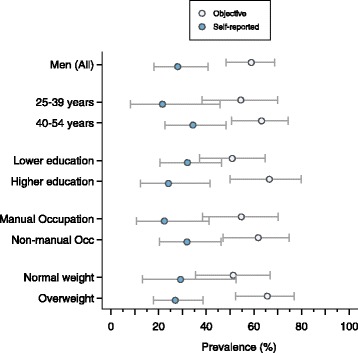
The prevalence of objective and self-reported physical inactivity in Barbadian men, stratified by sociodemographic group

For both genders, estimates of physical inactivity were underestimated by self-report compared with objective measures (Figs. 2 and 3; Additional file 3: Table S2). The prevalence (95 % CI) of self-reported inactivity was 64.9 % (56.3,72.7) in women and 27.7 % (17.6,40.6) in men. Physical inactivity was underestimated by the RPAQ by 25 percentage points (pp) in women (*p* value for difference: 0.001) and 31 pp (*p* value: 0.001) in men. The size of the difference between self-reported and objectively assessed inactivity tended to vary by sociodemographic characteristic, with statistically significant differences only in the older age groups, the more educated, those with non-manual occupations, and overweight/obese groups.

### Social distribution of physical activity using objective and self-reported measures

Table 4 presents the results of a median regression analysis to examine the distribution of objective PA components by indicators of SEP. Overall*,* after controlling for age and sex, objective PAEE, LPA, and MVPA were lower in people with more education and higher occupational grade, while sedentary time was higher. When men and women were examined separately, very few associations between PA parameters and SEP indicators were statistically significant, though the magnitude and direction of the relationships were similar to those found when examining men and women together.

**Table 4:** Distribution of objectively assessed physical activity components in young to middle aged adult Barbadians

	Women		Men		Overall	
	Coefficient	95 % CI	Coefficient	95 % CI	Coefficient	95 % CI
Education^a^						
PAEE (kJ/kg/day)	−0.9	(−3.9,2.1)	−4.2	(−9.7,1.3)	−1.8	(−4.6,−0.9)
Sedentary time (hours/day)	0.6	(0.1,1.0)	0.7	(0.2,1.3)	0.7	(0.3,1.1)
Time spent in light activity (hours/day)	−0.4	(−0.7,0.0)	−0.2	(−0.6,0.2)	−0.3	(−0.5,0.0)
Time spent in moderate-to-vigorous activity (minutes/day)	−6.7	(−15.5,2.2)	−19.5	(−40.8,1.7)	−10.2	(−19.9,−0.5)
Occupation^b^						
PAEE (kJ/kg/day)	−0.1	(−0.5,0.2)	−3.2	(−8.1,1.7)	−3.3	(−5.6,−1.0)
Sedentary time (hrs/day)	0.6	(0.1,1.0)	0.1	(−0.4,0.7)	0.5	(0.1,0.8)
Time spent in light activity (hrs/day)	−0.4	(−0.7,0.0)	−0.1	(−0.5,0.2)	−0.3	(−0.5,−0.1)
Time spent in moderate-to-vigorous activity (minutes/day)	−10.8	(−18.8,−2.7)	−18.0	(−36.2,−0.2)	−11.7	(−20.4,−3.0)

^a^Regression coefficient is the median change in the PA component as education category increases from lowest to highest, after controlling for age and sex. There are four education categories: level 1 had not completed secondary school; level 2 completed secondary school; level 3 had technical, trade or teacher education; and level 4 had university education (undergraduate and postgraduate)

^b^Regression coefficient is the median change in the PA component as occupation category increases from lowest to highest, after controlling for age and sex. There are three occupation groups : group 1 (routine/manual) consisted of skilled agricultural, craft/elementary workers, and machine operators; group 2 (intermediate) comprised technical, clerical and service employees; and group 3 (professional) consisted of managers and professionals

Using self-reported data, total PAEE was not associated with education, but domain-specific variation of energy expenditure by education was observed (data not shown in table). Education was found to be directly associated with self-reported leisure-time PAEE and inversely associated with self-reported occupational PAEE. For every increase in educational level, reported leisure-time PAEE was higher by 3.85 (95 % CI: 1.1,6.5) kJ/kg/day, and reported occupational PA was lower by 1.7 (0.1,3.3) kJ/kg/day.

### Self-reported measures: context and type of activity

The relative contribution of energy expenditure in different domains to total PAEE was examined using self-reported data (Additional file 3: Table S3), excluding unemployed participants (*n* = 78). Men and women had similar patterns of expenditure by domain. Occupational, leisure, home, and travel activity accounted for 67 %, 21 %, 18 %, and 4 % of total PAEE, respectively. Including unemployed participants did not change the pattern of domain-specific contributions to overall PAEE: occupational PAEE still made the largest contribution (45 % overall).

The most popular type of leisure activity for both sexes was walking, followed by gardening (Table 5). On average, women reported spending 60 min/week walking and 37 min/week gardening over the past four weeks. Men reported spending 54 min/week walking and 48 min/week gardening. Women reported close to no time participating in team sports, racket sports, or water sports, and very little time running, swimming or cycling (range: 3–13 min). Compared with women, men spent more time in these activities (range: 6–29 min), with the exception of water sports, which no men reported.

**Table 5:** Reported time spent carrying out leisure activities by category in young to middle aged adult Barbadians

	Women		Men	
	Mean time		Mean time	
	(minutes/week)	95 % CI	(minutes/week)	95 % CI
Walking	60	(35,85)	54	(26,82)
Gardening	37	(18,56)	48	(21,75)
Team sports	1	(−1,3)	29	(14,44)
Racquet sports	0	(0,0)	18	(1,36)
Running	3	(0,6)	10	(5,14)
Aerobics	14	(7,21)	7	(−4,19)
Swimming	3	(1,6)	23	(−11,57)
Cycling	2	(−2,6)	6	(0,12)
Water sports	1	(−1,3)	0	(0,0)

## Discussion

Our findings reveal a high prevalence of physical inactivity in this population: 90.5 % of women and 58.9 % of men did not accumulate sufficient activity to meet WHO minimum recommendations. Sedentary time is also likely to be a major contributor to NCD risk in this population: in both genders, median time spent sedentary was objectively estimated to be around 8 h per day, excluding sleeping. Furthermore, 60 % of women and men were classified as PA overestimators, i.e. they considered their activity to be sufficient though they were objectively classified as inactive. Self-reported measures underestimated the prevalence of inactivity by 25.5 pp in women and 31.3 pp in men. However, agreement between subjective and objective measures varied by age, education level, occupational grade, and BMI category. Low PA was higher in more socially privileged groups: higher educational attainment and higher occupational grade were both associated with less activity and more sedentary time. Occupational PA was the main driver of PAEE for women and men according to self-report. The most popular leisure time activities for both genders were walking, followed by gardening.

### Strengths and limitations

The main strength of our study was the use of objective and self-report measures of physical activity to provide complementary information in a nationally representative sample of a developing country population, where little is known about these behaviours, nationally and regionally. In this paper we provide the most comprehensive assessment of physical activity patterns in an adult, Caribbean population. However, before we consider the interpretation and importance of the findings it is important to acknowledge the limitations of the study.

We assessed adherence to the WHO recommendations for physical activity [29], which we interpreted as at least 150 min of MVPA each week. However, the guidelines also specify that muscle-strengthening activities should be carried out on at least 2 days a week. We were unable to assess this with the objective measure, and therefore did not consider it for either measure to ensure comparability. Our estimates of the prevalence of inactivity are therefore likely to be conservative.

To assign energy costs to the activities reported in the questionnaire, we used a published physical activity compendium [19], which does not take into account individual variation and variation between populations. We also assigned assumed energy costs for types of occupation (sedentary, standing, manual, and heavy manual), and these assumptions were based on measurements made in European populations. It is not clear whether average occupational energy expenditure differs in our population.

We examined domain-specific contribution to overall energy expenditure using RPAQ-derived measures. However, it is not clear whether the bias with which activity is reported differs by domain. Our assessment assumes equal bias, and could therefore be inaccurate.

Finally, we compared the prevalence of inactivity derived from an objective measure to that derived from a questionnaire. These assessments were made at different points in time, with a median of 114 days between them. However, it is unlikely that this gap affected the conclusions drawn from this comparison, which was made to determine the public health implications of using self-reported measures of inactivity at a population level. Although individual changes in activity may have occurred in the period between measurements, it is doubtful that there would have been meaningful changes in population levels of activity.

### Comparison with other studies

Our estimates of physical inactivity were high, but comparison with other populations is limited by different age profiles between populations and the application of different definitions of inactivity, as well as the use of different measurement techniques. In the US, the objective prevalence of inactivity was estimated as greater than 95 % overall [7], but this study used accelerometry and examined a different age range (20 years and over). Furthermore, the definition of inactivity used was based on a different implementation of the PA guidelines, with participants needing to accumulate 30 min of activity on at least 5 of 7 days of measurement [7]. As Thompson et al. [34] emphasize, even small changes in how inactivity is defined results in large variation in activity status. In the UK, 96 % of women and 94 % of men over the age of 16 years did not achieve the government’s recommended physical activity level, as assessed by accelerometry [35]. Again, this study examined a different age range, and used a different measurement technique. These important caveats aside, the level of inactivity observed in our population for women (90.5 %) was in a similar range to those reported for the US and UK, while the level of inactivity amongst men (58.5 %) was substantially lower. Estimates of population levels of inactivity in developing countries are based largely on questionnaires [6], and this limited our ability to draw meaningful comparisons in more similar settings. A study from Cameroon, also using combined heart rate and movement sensing, reported estimates of PAEE for rural and urban populations aged 25 to 55 years [36]. Mean PAEE in urban women and men was 37.9 and 51.5 kJ/kg/day, respectively, and 54.3 and 64.6 kJ/kg/day in rural women and men, respectively. Our estimates of PAEE in Barbadian women and men (36.4 and 47.2 kJ/kg/day, respectively) were similar to urban Cameroonian estimates, and less than rural Cameroonian estimates. In Kenya, similar methodology was used to measure PA in three rural populations [37]. The PAEE reported was substantially higher in all of these populations compared to our study, with the lowest (Luo population) being 58.9 and 74.4 kJ/kg/day in women and men, respectively.

In terms of age and gender patterns of PA, our data are consistent with a recent global review, which found that, on the whole, men are more physically active than women, and older people are less active than younger people [6]. Much less is known about the distribution of PA by SEP, even in developed countries. A recent European systematic review did not find consistent associations between total self-reported PA and SEP. [38] However, domain-specific gradients were reported: higher SEP was generally associated with more leisure-time activity and less occupational activity. The authors suggest that these findings demonstrate complex patterns of socioeconomic inequalities in physical activity, and that total activity may not be a suitable summary measure when investigating inequalities and how this affects morbidity and mortality. Our results imply that this complexity may be attributable to the use of questionnaires to measure activity, rather than a lack of social gradient for overall activity. Similar to European data, we observed a lack of association between overall self-reported total activity and education, whilst domain-specific associations exist in opposite directions for leisure-time and occupational activity. However, we also show that a clear social gradient in overall activity can be demonstrated when objective measures are used. The utility of questionnaires for describing social patterns of PAEE may therefore be limited, possibly due to social desirability bias.

Our finding that subjective methods substantially underestimate physical inactivity is consistent with results from the US [7] and the UK [8], and reinforces the need to interpret quantitative findings based on self-reported PA with care. A further note of caution comes from the difference in accuracy of PA reporting by age, SEP, and BMI category. Thus, while over-reporting of physical activity was apparent (based on the point estimates) in all the subgroups we examined (see Figs. 1 and 2) it was only statistically significant in in older age groups, the more educated, those with non-manual occupations, and overweight/obese groups. Over-reporting of PA by BMI category has been documented previously [39, 40]. Our results further underscore the need for objective measures to be used when investigating the relationship between physical activity and health outcomes that are related to BMI, as well as other demographic and socioeconomic characteristics. Another potential limitation of using questionnaires for PA surveillance is the possibility that objective and self-report measures differentially track trends over time, with people becoming more accustomed to giving socially desirable answers. If this is the case, trends in self-reported PA may represent bias in how the questionnaires are completed. Although this area remains largely unstudied, Cleland et al. [41] found that the Global Physical Activity Questionnaire (GPAQ) provided a valid measure of change in reported activity over time, compared with accelerometry. However, this study only repeated the measurements once, with a relatively short interval (3–6 months). It is therefore unlikely to represent a typical surveillance scenario, where large population-based surveys are repeated over years, with results publicised between rounds. Further studies that address this concern would help to fully determine the implications of using questionnaires for PA surveillance.

### Potential public health implications

This study demonstrates how combining objective and self-reported measures of PA and sedentary behaviours can provide useful information for guiding interventions in developing countries. The high prevalence of physical inactivity and having a sedentary lifestyle underscore the need for population-wide public health intervention. However, given the limited resources in this setting, a more pragmatic approach may be to target groups with particularly low activity. Our data highlight that women and those with a high SEP have particularly low levels of activity, and these needs should be considered when interventions are designed. A useful direction for future studies would be to utilise qualitative methodology to investigate how public health campaigns could effectively target the low-activity groups that we have identified. Alvarado et al. [42] have identified barriers to PA in young, overweight and obese Barbadian women, and have made recommendations on how activity could be facilitated in this group. Similar studies focusing on other low-activity groups are warranted.

In this population, 60 % of individuals overestimate their activity. This group is of particular public health importance, as people who believe they are sufficiently active are unlikely to see the need to increase their activity. The prevalence of PA overestimation in Barbados is higher than reported in a UK population, (46 %) [43], but similar to that reported in the Netherlands (61 %) [44]. Public education to improve awareness of PA levels should be considered an integral part of future efforts to increase activity.

Occupational PA is the main contributor to overall PAEE in this population, although this is to an extent driven by the assigned energy cost to the different occupations, combined perhaps with an underrepresentation of activities of daily living. Whether occupational PA is as beneficial for health as leisure PA is unclear [45], so this pattern may suggest that the energy is not being expended in a manner that optimises its impact on health. Active transport makes little to no contribution to overall PAEE (4 % for women and men). Anecdotally, a hot and humid climate, lack of changing facilities at work, an infra-structure of narrow roads with limited sidewalks, and a strong social preference for personal motorised transport are all cited as barriers to increasing levels of active transport in Barbados and similar Caribbean countries. Well designed, including qualitative, studies to properly investigate barriers to active transport and the feasibility of effectively promoting it are needed. A more promising focus for future interventions might be to encourage more leisure time activity. In other populations, higher socioeconomic groups tend to participate disproportionately more in leisure activities, compared with lower socioeconomic groups [38]. Encouraging leisure activities in these populations therefore has the potential to exacerbate social inequalities in physical activity. In the Barbadian population, however, we have demonstrated that physical inactivity is higher in those with a higher SEP, at least according to education and occupation. Population increases in leisure activity may therefore reduce PA inequality. Examining participation in different types of leisure activities highlights those that could be most effectively promoted. Walking was the most popular leisure activity, and interventions to increase population levels of walking have been successfully implemented in many populations [46]. Similar approaches could be adopted in our setting. An alternative strategy could be to identify gaps, and to promote currently uncommon activities. For example, on average, Barbadian women participate very little in team sports and racquet sports, with a mean time reported per week of less than 2 min. Efforts could be made to encourage participation of women in sports, perhaps starting with school-age girls. Further studies would be necessary to determine the how effective this approach is likely to be.

This study adds to a growing body of evidence that highlights the limitations of self-reported measures to assess inactivity prevalence. Despite this, questionnaires such as the GPAQ continue to be used in developing countries, and are widely integrated into national surveillance systems as the only instrument [47]. However, with objective methods becoming cheaper and more feasible to apply on scale, this may well change in the future. A stated target of WHO’s global action plan on non-communicable diseases is to reduce physical inactivity from a 2010 baseline by 10 % by 2025 [2]. We suggest that questionnaires on their own are not sufficiently valid for use in NCD risk factor surveillance systems, and their continued use in isolation impedes accurate evaluation of this important target. We emphasize the urgent need for cheaper and simpler objective methods for physical activity surveillance to be developed and implemented.

## Conclusions

Our findings demonstrate the high prevalence of physical inactivity in a small island developing country in the Caribbean. Physical inactivity is higher in women and the more socially privileged, and interventions to address these needs are warranted. Using questionnaires and objective methods together can provide information to guide and monitor public health programmes, but using questionnaires alone to derive quantitative assessments of physical inactivity will likely lead to spurious conclusions, both in terms of the levels of inactivity and their social distribution.

## Additional files

Additional file 1: Recent Physical Activity Questionnaire. (DOC 132 kb) Additional file 2: Summary Activities. (DOCX 14 kb) Additional file 3: Table S1. Participant characteristics of Health of the Nation sample aged 25–54 years. **Table S2.** Distribution of inactivity as assessed by objective and subjective measures for selected population characteristics. **Table S3.** Proportional contribution of each domain to total energy expenditure in young to middle aged Barbadian adults (excluding unemployed participants). (XLS 17 kb) Additional file 4: Information on sample recruitment and missing data. (DOCX 15 kb)

## Abbreviations

BMI: Body mass index
HotN: Health of the nation
IMF: International monetary fund
IQR: Interquartile range
LPA: Light physical activity
MET: Metabolic equivalent
MVPA: Moderate-to-vigorous physical activity
NCD: Non-communicable disease
PA: Physical activity
PAEE: Physical activity energy expenditure
Pp: Percentage points
SEP: Socioeconomic position
SIDs: Small Island Developing States
WHO: World Health Organisation
