# Supplementary material for: A cross-sectional study of physical activity and sedentary behaviours in a Caribbean population: combining objective and questionnaire data to guide future interventions
Source: BMC Public Health. 2016 Oct 1;16:1036. doi: 10.1186/s12889-016-3689-2 (PMC5045650; doi:10.1186/s12889-016-3689-2)
Supplement: Additional file 1: — Recent Physical Activity Questionnaire. (DOC 132 kb) [file 12889_2016_3689_MOESM1_ESM.doc]

**Participant study No.**

# RPAQ

## Recent Physical Activity Questionnaire

### This questionnaire is designed to find out about your physical activity in your everyday life in the last 4 weeks

**This questionnaire is divided into 3 sections**

### Please try to answer every question.

- **Section A** asks about your physical activity patterns in and around the house.
- **Section B** is about travel to work and your activity at work.
- **Section C** asks about recreations that you may have engaged in during the last 4 weeks.

#### Your answers will be treated as strictly confidential and will be used only for medical research

###### Section A Home Activities

Getting about

Which form of transport have you used **most often** in the last 4 weeks **apart from your journey to and from work?** (Please tick (✓) one box only)

| Usual mode of travel | | | |
| --- | --- | --- | --- |
| Car / motor vehicle | Walk | Public transport | Cycle |
|  |  |  |  |

TV, DVD or Video Viewing (Please put a tick (✓) on every line)

| Hours of TV, DVD or video watched per day | Average over the last 4 weeks | | | | | |
| --- | --- | --- | --- | --- | --- | --- |
|  | None | Less than 1 hour a day | 1 to 2 hours a day | 2 to 3 hours a day | 3 to 4 hours a day | More than 4 hours a day |
| On a weekday before 6 pm |  |  |  |  |  |  |
| On a weekday after 6 pm |  |  |  |  |  |  |
| On a weekend day before 6 pm |  |  |  |  |  |  |
| On a weekend day after 6 pm |  |  |  |  |  |  |

Computer use at home *but not at work* (e.g. internet, email, Playstation, Xbox, Gameboy etc) (Please put a tick (✓) on every line)

| Hours of home computer use per day | Average over the last 4 weeks | | | | | |
| --- | --- | --- | --- | --- | --- | --- |
|  | None | Less than 1 hour a day | 1 to 2 hours a day | 2 to 3 hours a day | 3 to 4 hours a day | More than 4 hours a day |
| On a weekday before 6 pm |  |  |  |  |  |  |
| On a weekday after 6 pm |  |  |  |  |  |  |
| On a weekend day before 6 pm |  |  |  |  |  |  |
| On a weekend day after 6 pm |  |  |  |  |  |  |

Stair climbing at home (please put a tick (✓) on every line)

| Number of times you climbed up a flight of stairs (approx 10 steps) each day at home | Average over the last 4 weeks | | | | | |
| --- | --- | --- | --- | --- | --- | --- |
|  | None | 1 to 5 times a day | 6 to 10 times a day | 11 to 15 times a day | 16 to 20 times a day | More than 20 times a day |
| On a weekday |  |  |  |  |  |  |
| On a weekend day |  |  |  |  |  |  |

Please answer this section to describe if you have been in paid employment at any time **during the last 4 weeks** or you have done regular, organised voluntary work.

Have you been in employment during the last 4 weeks? *Yes No*

During each of the last 4 weeks how many hours work did you do per week?

|  | 1 weeks ago | 2 weeks ago | 3 weeks ago | 4 week ago |
| --- | --- | --- | --- | --- |
| Work hours  (excluding travel) |  |  |  |  |

Type of work

We would like to know the type and amount of physical activity involved in your work. **Please tick** (✓) the option that **best** corresponds with your occupation(s) in the last 4 weeks from the following four possibilities:

***Please tick only one of the following***

1. Sedentary occupation

You spend most of your time sitting (such as in an office)

1. Standing occupation

You spend most of your time standing or walking. However, your work does

not require intense physical effort (e.g. shop assistant, hairdresser, guard)

1. **Manual work**

##### This involves some physical effort including handling of heavy objects

##### and use of tools (e.g. plumber, electrician, carpenter)

1. **Heavy manual work**

##### This implies very vigorous physical activity including handling of very

##### heavy objects (e.g. dock worker, miner, bricklayer, construction worker)

##### **Travel to and from work in the last 4 weeks**

##### How many times a week did you travel from home to your main work?

##### *Count outward journeys only*

##### Please tick () one box **only** per line

| **How did you normally travel to work?** | Always | Usually | Occasionally | Never or rarely |
| --- | --- | --- | --- | --- |
| By car/motor vehicle |  |  |  |  |
| By works or public transport |  |  |  |  |
| By bicycle |  |  |  |  |
| Walking |  |  |  |  |

Work address (we need this to calculate distance between your home and work) –

____________________________

____________________________

____________________________

The following questions ask about how you spent your leisure time.

Please indicate how often you did each activity on average over the last 4 weeks

Please indicate the average length of time that you spent doing the activity on each occasion.

**Example**

If you went walking for pleasure for 40 minutes once a week.

If you had done weeding or pruning every fortnight and took 1 hour and 10 minutes on each occasion.

You would complete the table below as follows:

**Please give an answer for the NUMBER OF TIMES you did the following activities in the past 4 weeks and the AVERAGE TIME you spent on each activity.**

**Please complete EACH line**

|  | Number of times you did the **activity in the last 4 weeks** | | | | | | | Average time per episode | |
| --- | --- | --- | --- | --- | --- | --- | --- | --- | --- |
|  | None | Once in the last 4 weeks | 2 to 3 times in the last 4 weeks | Once a week | 2 to 3 times a week | 4 to 5 times a week | Every day | Hours | Minutes |
| Weeding and pruning |  |  | ✓ |  |  |  |  | *1* | *10* |
| Walking for pleasure |  |  |  | ✓ |  |  |  |  | *40* |

Now complete the table on pages 6 and 7

**Please give an answer for the average time you spent** on each activity and the number of times you did that activity in the past 4 weeks

**Please complete each line**

|  | Number of times you did the **activity in the last 4 weeks** | | | | | | | | Average time per episode | |
| --- | --- | --- | --- | --- | --- | --- | --- | --- | --- | --- |
|  | None | Once in the last 4 weeks | 2 to 3 times in the last 4 weeks | Once a week | | 2 to 3 times a week | 4 to 5 times a week | Every day | Hours | Minutes |
| Swimming -competitive |  |  |  |  | |  |  |  |  |  |
| Swimming leisurely |  |  |  |  | |  |  |  |  |  |
| Swimming for exercise (e.g. laps) |  |  |  |  | |  |  |  |  |  |
| Hiking |  |  |  |  |  | |  |  |  |  |
| Walking for exercise (including treadmill) |  |  |  |  |  | |  |  |  |  |
| Walking for pleasure *(not as a means of transport)* |  |  |  |  |  | |  |  |  |  |
| Racing or rough terrain cycling |  |  |  |  |  | |  |  |  |  |
| Cycling for pleasure *(not as a means of transport)* |  |  |  |  |  | |  |  |  |  |
| Mowing the lawn |  |  |  |  |  | |  |  |  |  |
| Watering the lawn or garden |  |  |  |  |  | |  |  |  |  |
| Digging, shovelling or chopping wood |  |  |  |  |  | |  |  |  |  |
| Weeding or pruning |  |  |  |  |  | |  |  |  |  |
| Maintenance around the home or car maintenance (including car washing) |  |  |  |  |  | |  |  |  |  |
| High impact aerobics or step aerobics |  |  |  |  |  | |  |  |  |  |
| Other types of aerobics |  |  |  |  |  | |  |  |  |  |
| Exercise with weights |  |  |  |  |  | |  |  |  |  |
| Conditioning exercises e.g. using a bike or rowing machine |  |  |  |  |  | |  |  |  |  |
| Push-ups, sit-ups or pull-ups |  |  |  |  |  | |  |  |  |  |

**Please complete each line**

|  | Number of times you did the **activity in the last 4 weeks** | | | | | | | Average time per episode | | |
| --- | --- | --- | --- | --- | --- | --- | --- | --- | --- | --- |
|  | None | Once in the last 4 weeks | 2 to 3 times in the last 4 weeks | Once a week | 2 to 3 times a week | 4 to 5 times a week | Every day | Hours | | Minutes |
| Floor exercises e.g. stretching, bending, keep fit or yoga |  |  |  |  |  |  |  |  | |  |
| Dancing |  |  |  |  |  |  |  |  | |  |
| Competitive running |  |  |  |  |  |  |  |  | |  |
| Jogging |  |  |  |  |  |  |  |  | |  |
| Tennis or badminton |  |  |  |  |  |  |  |  | |  |
| Squash |  |  |  |  |  |  |  |  | |  |
| Table tennis |  |  |  |  |  |  |  |  | |  |
| Road tennis or paddle ball |  |  |  |  |  |  |  |  | |  |
| Golf |  |  |  |  |  |  |  |  | |  |
| Football rugby or hockey |  |  |  |  |  |  |  |  | |  |
| Cricket |  |  |  |  |  |  |  |  | |  |
| Rowing, kayaking or stand-up paddle boarding |  |  |  |  |  |  |  |  | |  |
| Netball, volleyball or basketball |  |  |  |  |  |  |  |  | |  |
| Fishing |  |  |  |  |  |  |  |  | |  |
| Horse-riding |  |  |  |  |  |  |  |  | |  |
| Musical instrument playing or singing |  |  |  |  |  |  |  |  |  | |
| Sailing, wind-surfing, kite surfing or boating |  |  |  |  |  |  |  |  |  | |
| Surfing or boogie boarding |  |  |  |  |  |  |  |  |  | |
| Martial arts, boxing or wrestling |  |  |  |  |  |  |  |  |  | |
| Child-minding |  |  |  |  |  |  |  |  |  | |

**Thank you.**
