# Supplementary material for: A cross-sectional study of physical activity and sedentary behaviours in a Caribbean population: combining objective and questionnaire data to guide future interventions
Source: BMC Public Health. 2016 Oct 1;16:1036. doi: 10.1186/s12889-016-3689-2 (PMC5045650; doi:10.1186/s12889-016-3689-2)
Supplement: Additional file 2: — Summary Activities. (DOCX 14 kb) [file 12889_2016_3689_MOESM2_ESM.docx]

Summary Activities: RPAQ

1. Swimming: leisure swimming, competitive swimming, and swimming for exercise
2. Walking: hiking, walking for exercise, and walking for pleasure
3. Running: competitive running and jogging
4. Cycling: leisure cycling and racing cycling
5. Aerobics: high impact aerobics, step aerobics, and other types of aerobics
6. Gardening: mowing the lawn, watering the lawn or garden, digging, shoveling, chopping wood, and weeding or pruning
7. Racquet sports: tennis, badminton, squash, table tennis, road tennis, and paddle ball
8. Water sports: rowing, kayaking, paddle boarding, sailing, wind-surfing, kite surfing, boating, surfing, boogie boarding
9. Team sports: football, rugby, hockey, cricket, netball, volleyball, and basketball
