# Supplementary material for: A cross-sectional study of physical activity and sedentary behaviours in a Caribbean population: combining objective and questionnaire data to guide future interventions
Source: BMC Public Health. 2016 Oct 1;16:1036. doi: 10.1186/s12889-016-3689-2 (PMC5045650; doi:10.1186/s12889-016-3689-2)
Supplement: Additional file 4: — Information on sample recruitment and missing data. (DOCX 15 kb) [file 12889_2016_3689_MOESM4_ESM.docx]

**Information on sample recruitment and missing data**

**Recruitment**

This physical activity study was nested within a larger risk factor survey (Health of the Nation; HotN). All HotN participants in the target age range were invited to take part in the physical activity study. Participants were excluded if they reported any of the conditions, which would preclude normal activity:

1. Aortic aneurysm
2. Aortic stenosis
3. Crescendo angina
4. Recent myocardial infarction (which has occurred within the previous 3 months or where the participant has not yet been approved for exercise by a physician)
5. Myocarditis
6. Pulmonary or systemic embolism within the last 4 weeks
7. Cardiomyopathy
8. Unable to walk unaided for a minimum of 10-minutes
9. Pregnancy: pregnant women were excluded due to differences in physical activity recommendations and also difficulties with exercise testing

We compared key characteristics (table 1) of the physical activity sample to the main study sample (supplementary table 1) to determine whether selection of physical activity participants was biased. There were no significant differences (with all 95% CIs overlapping) between the HotN sample and the physical activity sample.

**Missing data**

All questionnaire data was checked when the questionnaires were completed. If any data points were missing, the data collector contacted the participant to retrieve. There were therefore no missing data points from the questionnaires at the analysis stage.

For the objective data, technical faults with the monitor occurred in six cases. These participants were contacted to determine whether they would wear the monitor again; however, all refused. These participants were excluded from the analysis. In addition, four participants wore the monitor for less than 24 hours; these data were excluded from the analysis.
